# Synthesis and Physico-Chemical Properties of New Tetraethylammonium-Based Amino Acid Chiral Ionic Liquids

**DOI:** 10.3390/molecules15042388

**Published:** 2010-04-05

**Authors:** Mohd Basyaruddin Abdul Rahman, Khairulazhar Jumbri, Mahiran Basri, Emilia Abdulmalek, Kamaliah Sirat, Abu Bakar Salleh

**Affiliations:** 1 Department of Chemistry, Faculty of Science, Universiti Putra Malaysia, 43400 UPM Serdang, Selangor, Malaysia; E-Mails: khairulazhar_evo@yahoo.com (K.J.); mahiran@science.upm.edu.my (M.B.); emilia@science.upm.edu.my (E.A.); kamaliah@fsas.upm.edu.my (K.S.); 2 Structural Biology Research Centre, Malaysia Genome Institute, UKM-MTDC Smart Technology Centre, 43600 UKM Bangi, Selangor, Malaysia; 3 Laboratory of Industrial Biotechnology, Institute of Bioscience, Universiti Putra Malaysia, 43400 UPM Serdang, Selangor, Malaysia; E-Mail: abubakar@biotech.upm.edu.my (A.B.S.)

**Keywords:** chiral ionic liquids, tetraethylammonium, amino acids, viscosity, ionic conductivity

## Abstract

This paper reports the synthesis of a series of new tetraethylammonium-based amino acid chiral ionic liquids (CILs). Their physico-chemical properties, including melting point, thermal stability, viscosity and ionic conductivity, have been comprehensively studied. The obtained results indicated that the decomposition for these salts proceeds in one step and the temperature of decomposition (*T*_onset_) is in the range of 168–210 °C. Several new CILs prepared in this work showed high ionic conductivity compared to the amino acid ionic liquids (AAILs) found in the literature.

## Abbreviations

[N_2222_]: tetraethylammonium
[ser]: serinate
[pro]: prolinate
[thr]: threoninate
[ile]: isoleucinate
[asn]: asparaginate
[gln]: glutaminate
[glu]: glutamate
[met]: methioninate
[his]: histidinate

## 1. Introduction

Ionic liquids (ILs), molten salts below 100 °C, have gained a lot of attention in the science and industry communities as reaction media, extraction solvents, electrolytes and biocatalysts [1]. These liquids contain ions which show good and tunable solubility properties combined with negligible vapour pressure and excellent thermal stability. At the same time, they have found a place of choice as valuable substitutes for many volatile solvents [2,3], which can dissolve polar to non-polar substrates and almost anything including coal, plastics, woods and even rocks [4]. They are not as flammable as classical volatile organic solvents (VOSs), therefore making the processes safer and environmental concerns less of an issue. Thermodynamic and kinetics of reactions carried out in these liquids could be different to those in traditional solvents, which has led to great interest in their potential use as solvents and co-solvents amongst chemists.

In the last few years, researchers have turned their interest to synthesis of chiral ionic liquids (CILs). Many CILs have been designed, synthesized and used in organic reactions. For example, a few researchers have reported that CILs have great potential applications as reaction media in chiral discrimination, asymmetric synthesis, and optical resolution of racemates [5,6,7]. Chiral ionic liquids can be synthesized from readily available starting materials containing chiral anions/cations or by using modification of non-chiral materials to produce chiral products. However, the modification reactions are usually more complicated and many steps are required to complete the whole reaction. By using starting materials such as lactates, sugars, sugar substitutes, plant acids and amino acids, a variety of CILs can be produced.

Recently, we have reported the synthesis of new tetraethylammonium-based CILs derived from plant acids [8,9]. As part of our ongoing study, amino acids have now been selected as starting material to produce CILs since they have a carboxylic acid residue, an amino group and a side chain that varies between different amino acids. Until now, a fairly large number of novel CILs based on amino acid cations or anions have been prepared [10,11,12,13,14,15,16], but most of them have relatively low ionic conductivities, higher viscosities and melting points. This viscous characteristic of amino acid ionic liquids (AAILs) limits many of their potential applications as fluids and electrolyte materials. The strong intermolecular interactions especially H–bonding in the AAILs was supposed to be the key point for the high melting points and viscosities [14,17]. Some literatures have reported that AAILs containing longer alkyl chains such as tetrabutylammonium, tetrabutylphosphonium and dialkyl-imidazolium show high viscosity and low conductivity at room temperature [13,19]. In this work, we aimed to study the effect of shorter alkyl chain of the tetraethylammonium cation, especially with regard to their thermodynamic properties of the resulting AAILs. We expect that the shorter alkyl chain of this cation will reduce the viscosity and increase the ionic conductivity.

## 2. Results and Discussion

### 2.1. Synthesis and Characterization

**Scheme 1:**
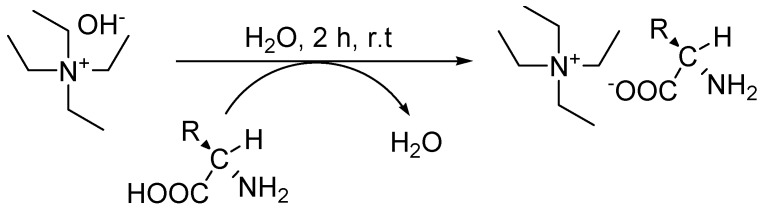
Synthetic pathway to tetraethylammonium-based chiral ionic liquids.

New tetraethylammonium-based amino acids CILs were prepared according to a method modified from literature [16]. A simple and straightforward synthetic pathway involved neutralization of commercially available starting materials that gave CILs in good overall yield (>85%), as shown in Scheme 1.

This method has an advantage since the by-product is only water, thus avoiding the use of metal salts and ion-exchange. Therefore, the purification of the CILs only required the removal of water. Most of the IL preparations have been carried out by formation of an organic halide salts which result in halide contamination. Subsequent separation of the halide can also post difficulties and give impurities that can change the properties of the desired ILs [18]. Therefore, in this work, halide contamination and extensive of purification procedures were avoided. The reactions were carried out at room temperature because tetraethylammonium hydroxide can readily deprotonate the carboxylic acid moiety of amino acids.

### 2.2. Thermal Stability and Physico-Chemical Properties

**Figure 1:**
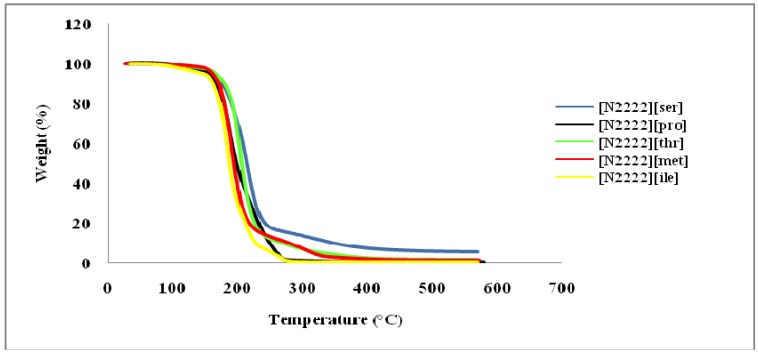
Thermogravimetric analysis of several new tetraethylammonium-based amino acids chiral ionic liquids.

The melting point (*T*_m_), decomposition temperature (*T*_onset_), viscosity (*η*) and ionic conductivity (*κ*) data of the prepared CILs are summarized in Table 1. They are thermally stable up to 160–210 °C (Figure 1), which is low compared to alkylimidazolium based-ILs (general *T*_d _> 300 °C). Interestingly, the decomposition temperature of these salts was a little lower than those of 1-ethyl-3-methyl-imidazolium-based ([emim][amino acids]) ILs (*T*_d_ around 220 °C) [13] and tetrabutylphosphonium-based ([TBP][amino acids]; *T*_d_ 210–320 °C) [19]. Although it had not been proven, we suspect that, the lower observed *T*_onset _of our CILs are the result of *Hoffman* elimination reaction of tetraethylammonium salt.

**Scheme 2:**
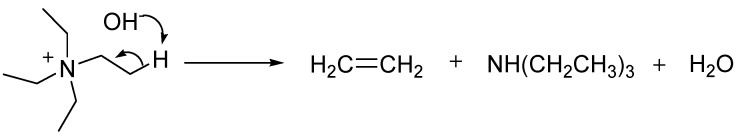
*Hoffman* elimination reaction.

A schematic presentation of this decomposition path is shown in Scheme 2. As it was discussed previously by MacFarlane *et al.*, [20], quaternary ammonium salts in a basic environment (ionized amino acids are slightly basic) are subject to a decomposition mechanism related to *Hoffman* elimination. Meanwhile, changing the amino acids anions had little effect on thermal stability.

All of these new compounds can be classified as ILs since their *T*_m_ are below 100 °C. The main factor that influences the *T*_m_ of these CILs is the structure of the corresponding anions. The two solid CILs, which are [N_2222_][asn] and [N_2222_][his], show *T*_m_ values of 58 °C and 54 °C, respectively. In comparison to [his], [asn] has a more symmetrical structure that may have attributed to its higher *T*_m_. As reported by Jiang *et al.* [16], the high degree of asymmetric structure of amino acids contributed to the low *T*_m _values of tetraalkylammonium-based amino acids ILs. However, the higher *T*_m _of these two solid CILs than the rest of CILs prepared cannot be fully explained. We are currently in the process of obtaining the corresponding crystal structure data to ascertain the packing structure of the salts.

**Table 1:** The physico-chemical properties of tetraethylammonium-based chiral ionic liquids.

No	CILs	*T*_m_ (°C)	*T*_onset_ (°C)	*η* (cP)	*κ* (mS cm^-1^)
1	[N_2222_][ser]	ND	187	1763	0.16
2	[N_2222_][pro]	ND	172	438	0.46
3	[N_2222_][thr]	ND	188	1002	0.24
4	[N_2222_][ile]	ND	168	526	0.35
5	[N_2222_][asn]	58 ± 1.0	191	a	a
6	[N_2222_][gln]	ND	210	a	a
7	[N_2222_][glu]	ND	210	a	a
8	[N_2222_][met]	ND	176	462	0.45
9	[N_2222_][his]	54 ± 1.0	174	a	a

ND: not detected; a: solid or glass at 25 °C, hence the property was not determined

### 2.3. Viscosity (η) and Ionic Conductivity (κ)

**Figure 2:**
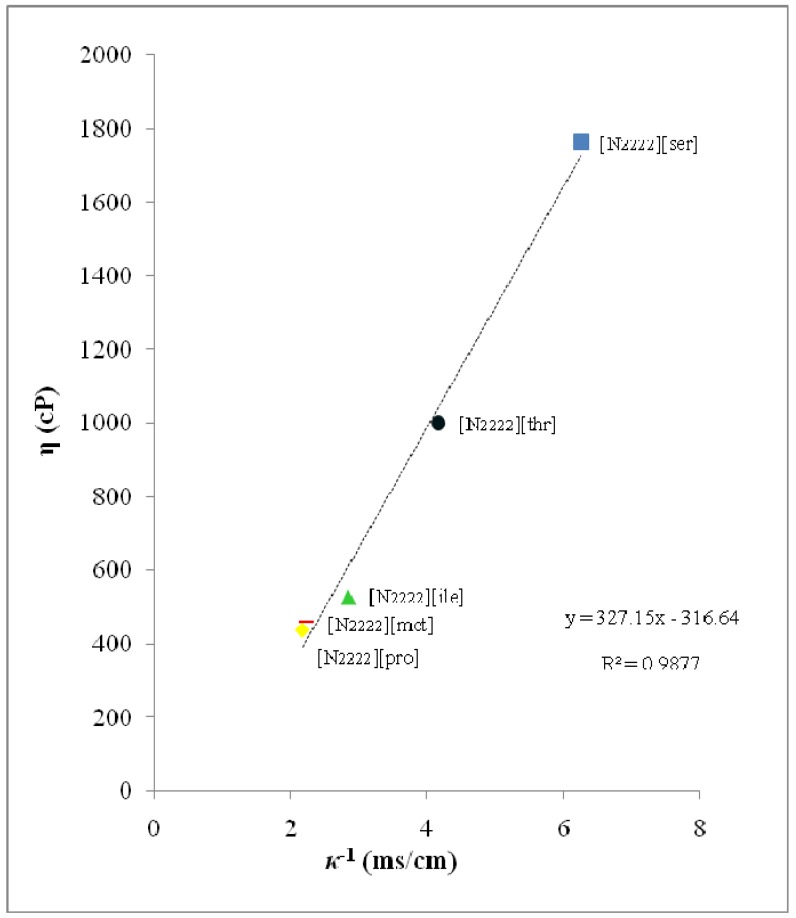
Relationship between ionic conductivity (*κ*^-1^) and viscosity (*η*) for five tetraethylammonium-based chiral ionic liquids at 25 °C.

Thermodynamic properties such as viscosity (*η*) and ionic conductivity (*κ*) of these new salts which are liquids at room temperature were measured and the corresponding data are presented in Table 1. According to the results, these salts showed conductivity values ranged from 0.16–0.54 ms cm^-1^, were much higher than those of similar ILs, [emim][amino acids] (*κ* = 9.1 × 10^-6 ^to 0.65 ms cm^-1^) [13]. In this work, [N_2222_][pro] displayed the highest ionic conductivity (0.46 ms cm^-1^) – three times higher than [emim][pro] (0.16 ms cm^-1^) – while [N_2222_][ser] (0.16 ms cm^-1^) showed the lowest ionic conductivity among [N_2222_][amino acids]. The lower conductivity of [emim] was due to the protons at the 2, 4 and 5 positions that contribute to the H-bonding interaction [13]. This interaction becomes the reason of low ionic conductivity of [emim][amino acids] as compared to our CILs. For the same cation, the ionic conductivities values of anion are in the following order: [ser] < [thr] < [ile] < [met] < [pro]. We found that the ionic conductivity of these salts closely related to the viscosity. In this study, the salts followed the linear relationship between ionic conductivity and viscosity (Figure 2). 

This linear correlation indicated that at 25 °C, the conductivity value is strongly affected by viscosity, and thus the salts with low viscosity showed high ionic conductivities. This may be due to an increase in the ion mobility of the salts. The cation structure strongly influences the viscosity, which is governed essentially by *van der Waals* interactions and H–bonding ability. It can be seen that, the [N_2222_] structure has a much small molecular weight as well as short and flexible alkyl chain. The short alkyl chains made the salts less viscous due to a decrease in *van der Waals* interactions and increase rate of ion mobility of the salts ensuing the high ionic conductivity. 

However, these new salts still showed higher viscosity than conventional ILs. This phenomenon happens due to the symmetry properties of the cation structure. Generally, symmetric cations usually bring high viscosity of aliphatic ammonium-based ILs [21,22]. Furthermore, the side chain of the corresponding amino acids and H–bonding interaction in the structure of ILs were also affect the viscosity and ionic conductivity [13]. H-bonding interaction should increase the solution viscosity as well as decrease the ionic conductivity. Especially in the case of amino acids as anion, most of them contain functional group such as not only amino group, but also carboxyl, hydroxyl and so on. This implies some intra- and inter-molecular interaction influenced in the packing structure of CILs. In others word, introduction of functional groups such as H-bonding donor or acceptor increased the viscosity and decreased the ionic conductivity through intra/intermolecular interaction [1].

For example, the salts [N_2222_][ser] (*η* = 1763 cP) and [N_2222_][thr] (*η* = 1002 cP) have relatively high viscosity that may be due to H–bonding or some other interaction which were expected through the side chains. The presence of hydroxyl group in both anions allowed the molecule to form H–bonding through inter/intramolecular interaction. Thus, the existences of strong H–bonding cause high viscosity and low ionic conductivity of these CILs. However, the relationship between the component ion structures with viscosity and ionic conductivity is not fully understood probably because there are several parameters, such as shape of ions, charge density and conformational changes of alkyl chain that involve in the structure of ILs [23].

## 3. Experimental

### 3.1. General

All chemicals were commercially available and of analytical grade unless otherwise specified. ^1^H- and ^13^C-NMR spectra were recorded with JEOL JNM-ECA 400 MHz and chemical shifts δ (in ppm) were related to residual solvent signal (D_2_O). Coupling constant *J* is expressed in Hz. Decomposition temperatures were measured with a Mettler-Toledo TGA/SDTA 851^e^ Thermal Gravimetric Analyzer (TGA) instrument under N_2_ atmosphere. The Mettler-Toledo STAR^e^ software version 8.10 was used to determine the onset temperature (*T*_onset_). All samples were run in alumina pan at heating rate 10 °C min^-1^ and the temperature was programmed from 25–600 °C. Melting points (*T*_m_) were determined by using Mettler-Toledo Differential Scanning Calorimeter (DSC), model DSC822^e^ and data were evaluated using Mettler-Toledo STAR^e^ software version 9.01. Measurement was carried out at a scan rate of 10 °C min^-1^ by heating and cooling the samples from −60–130 °C. Viscosity (*η*) was measured at 25 °C using Brookfield LVDV-II+Pro cone/plate viscometer equipped with CPE-52 spindle. The instrument was connected to computer with Rheocalc32 software version 3.1. Ionic conductivity (*κ*) was determined at 25 °C by using SevenGo Conductivity Meter. Measurements of optical rotation were carried out using Jasco P-200 Polarimeter equipped with a 100 mm standard glass tube with bulb at 25 °C. The data were recorded on instruments which use the sodium lamp as a radiation source and wavelength is constant and standard. The data was then recorded three times and the average was taken as an observed optical rotation value (α). The value was then used in the formula (1) to calculate the specific optical rotation value, [α]_D_^25^:

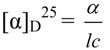
(1)
where [α] = specific optical rotation; *α* = value of optical rotation observed; *l* = length of cell (dm); *c* = concentration in g/100 mL.

### 3.2. Typical Synthetic Procedure for Tetraethylammonium-Based Amino Acids

An aqueous solution of tetraethylammonium hydroxide (20% w/w, 50 mmol) was added to a solution of excess amino acid (50 mmol). The reaction mixture was stirred for 2 h at room temperature. Then, the crude products were obtained after being dried at 70 °C under vacuum for 1 day. Since the crude product was miscible with EtOH and amino acid was nearly insoluble in EtOH, the crude product was added into EtOH (50 mL) to precipitate the excess of amino acid. After filtration, the solvent was evaporated and the product was obtained after being dried in *vacuo* for 1 day at 65 °C.

*Tetraethylammonium* *L-serinate ([N_2222_][ser])*: Yield 96%; [α]_D_^25 ^= -1.5 (*c* 1, H_2_O); ^1^H-NMR δ 3.82–3.77 (m, 2H, HO-C*H_2_*), 3.39–3.20 (m, 1H, O_2_C-C*H*), 3.25 (q, ^3^*J*_HH_ = 7.3 Hz, 8H, N-C*H_2_*), 1.25 (t, ^3^*J*_HH_ = 7.3 Hz, 12H, N-CH_2_-C*H_3_*); ^13^C-NMR δ 6.77, 52.03, 57.55, 64.16, 179.02; Anal. calcd. for C_11_H_26_N_2_O_3_; C 56.38, H 11.18, N 11.95; found: C 56.00, H 11.01, N 11.48. 

*Tetraethylammonium* *L-prolinate ([N_2222_][pro])*: Yield 90%; [α]_D_^25^ = -42.9 (*c* 1, H_2_O); ^1^H-NMR δ 3.45 (dd, 1H, ^3^*J*_H1Hα _= 7.3 Hz, ^3^*J*_H1Hβ _= 12.12 Hz, O_2_C-C*H*), 3.12-3.09 (m, 1H, N-C*H*(α)), 3.21 (q, ^3^*J*_HH_ = 7.3 Hz, 8H, N-C*H_2_*), 2.99–2.74 (m, 1H, N‑C*H*(β)), 2.17–2.03 (m, 1H, HC-C*H*(α)), 1.72 to 1.61 (m, 3H, HC-C*H*(β) and H_2_N-CH_2_-C*H_2_*), 1.14 (t, ^3^*J*_HH_ = 7.3 Hz, 12H, N-CH_2_-C*H_3_*); ^13^C-NMR δ 6.75, 25.10, 30.67, 46.19, 51.96, 61.56, 180.28; Anal. calcd. for C_13_H_28_N_2_O_2_: C 63.89, H 11.55, N 11.46; found: C 63.53, H 11.23, N 11.07. 

*Tetraethylammonium* *L-threoninate ([N_2222_][thr])*: Yield 94%; [α]_D_^25^ = -3.8 (*c* 1, H_2_O); ^1^H-NMR δ 3.82–3.77 (m, 1H, OH-C*H*), 3.14 (q, ^3^*J*_HH_ = 7.5 Hz, 8H, N-C*H_2_*), 2.94 (d, ^3^*J*_HH_ = 5.5 Hz, 1H, O_2_C-C*H*), 1.13 (t, ^3^*J*_HH_ = 7.5 Hz, 12H, N-CH_2_-C*H_3_*), 1.06 (d, ^3^*J*_HH_ = 6.0 Hz, 3H, OH-C-C*H_3_*); ^13^C-NMR 6.58, 19.33, 57.73, 68.16, 70.54, 180.32; Anal. calcd. for C_12_H_28_N_2_O_3_: C 58.03, H 11.36, N 11.28; found: C 57.90, 11.14, N 10.97.

*Tetraethylammonium* *L-isoleucinate ([N_2222_][ile])*: Yield 87%; [α]_D_^25^ = +4.9 (*c* 1, H_2_O); ^1^H-NMR δ 3.13 (q, ^3^*J*_HH_ = 7.3 Hz, 8H, N-C*H_2_*), 2.96 (d, ^3^*J*_HH_ = 5.0 Hz, 1H, O_2_C-C*H*), 1.65–1.54 (m, 1H, H_2_N-CH-C*H*), 1.30–1.28 (m, 2H, CH-C*H_2_*), 1.13 (t, ^3^*J*_HH_= 7.3 Hz, 12H, N-CH_2_-C*H_3_*), 1.17–1.01 (m, 3H, CHC*H_3_*), 0.82–0.76 (m, 3H, CH_2_C*H_3_*); ^13^C-NMR δ; 6.63, 11.38, 15.61, 25.19, 39.74, 60.43, 57.81, 179.27; Anal. calcd. for C_14_H_32_N_2_O_2_: C 64.57, H 12.39, N 9.89; found: C 64.25, H 12.24, N 9.76.

*Tetraethylammonium L-asparaginate ([N_2222_][asn])*: Yield 92%; [α]_D_^25^ = -5.1 (*c* 1, H_2_O); ^1^H-NMR δ 3.69–3.57 (m, 1H, O_2_C-C*H*), 3.25 (q, ^3^*J*_HH_ = 7.5 Hz, 8H, N-C*H_2_*), 2.64 (dd, ^2^*J*_HαHβ_ = 12.5 Hz, ^3^*J*_HH _= 5.5 Hz, 1H, OOC-C-C*H*(α)), 2.42 (dd, ^2^*J*_HαHβ _= 12.5 Hz, ^3^*J*_HH_ = 8.0 Hz, 1H, OOC-C-C*H*(β)), 1.26 (t, ^3^*J*_HH_ = 7.5 Hz, 12H, N-CH_2_-C*H_3_*); ^13^C-NMR δ 6.75, 40.20, 55.21, 57.10, 176.32, 179.90; Anal. calcd. for C_12_H_27_N_3_O_3_: C 55.15, H 10.41, 16.08; found: C 54.93, H 10.16, N 15.83. 

*Tetraethylammonium* *L-glutaminate ([N_2222_][gln])*: Yield 91%; [α]_D_^25^ = -7.4 (*c* 1, H_2_O); ^1^H-NMR δ 3.96 (t, ^3^*J*_HH_ = 7.3 Hz, 1H, O_2_C-C*H*), 3.05 (q, ^3^*J*_HH_ = 7.3 Hz, 8H, N-C*H_2_*), 2.35 to 2.14 (m, 2H, CH-CH_2_-C*H_2_*), 1.88 to 1.61 (m, 2H, CH-C*H_2_*-), 1.08 (t, ^3^*J*_HH_ = 7.3 Hz, 12H, N-CH_2_-C*H_3_*); ^13^C-NMR δ 6.72, 30.39, 33.84, 56.75, 58.51, 176.43, 181.95; Anal. calcd. for C_13_H_29_N_3_O_3_: C 56.70, H 10.61, N 15.26; found: C 56.38, H 10.34, N 15.04. 

*Tetraethylammonium* *L-glutamate ([N_2222_][glu])*: Yield 89%; [α]_D_^25^ = -2.1 (*c* 1, H_2_O); ^1^H-NMR δ 3.74–3.58 (m, 1H, O_2_C-C*H*), 3.24 (q, ^3^*J*_HH_ = 7.3 Hz, 8H, N-C*H_2_*), 2.57-2.33 (m, 2H, O_2_-C*H_2_*-), 2.16–1.99 (m, 2H, O_2_-CH_2_-C*H_2_*), 1.25 (t, ^3^*J*_HH_ = 7.3 Hz, 12H, N-CH_2_-C*H_3_*); ^13^C-NMR δ 6.70, 27.12, 33.62, 51.95, 54.68, 174.40, 180.94; Anal. calcd. for C_13_H_28_N_2_O_4_: C 56.50, H 10.21, N 10.14; found: C 56.27, H 10.04, N 9.96. 

*Tetraethylammonium* *L-methioninate ([N_2222_][met])*: Yield 95%; [α]_D_^25^ = +1.1 (*c* 1, H_2_O); ^1^H-NMR δ 3.49–3.36 (m, 1H, O_2_C-C*H*), 3.25 (q, ^3^*J*_HH_ = 7.3 Hz, 8H, N-C*H_2_*), 2.51 (t, ^3^*J*_HH_ = 8 Hz, 2H, S-C*H_2_*), 2.11 (s, 3H, S-C*H_3_*), 1.82 to 1.99 (m, 2H, CH-C*H_2_*), 1.25 (t, ^3^*J*_HH_ = 7.3 Hz, 12H, N-CH_2_-C*H_3_*); ^13^C-NMR δ 6.82, 14.35, 29.87, 33.90, 52.05, 55.30, 181.52; Anal. calcd. for C_13_H_30_N_2_O_2_S: C 56.07, H 10.86, N 10.06, S 11.49; found: C 55.87, H 10.64, N 9.88, S 11.18. 

*Tetraethylammonium* *L-histidinate ([N_2222_][his])*: Yield 86%; [α]_D_^25^ = -5.2 (*c* 1, H_2_O); ^1^H-NMR δ 7.65 (s, 1H, N=C*H*-NH), 6.91 (s, 1H, HN-C*H*=C), 3.49 (t, ^3^*J*_HH _= 7.3 Hz, 1H, O_2_C-C*H*), 3.19 (q, ^3^*J*_HH_ = 7.3 Hz, 8H, N-C*H_2_*), 2.97 (dd, ^2^*J*_HαHβ_ = 12.5 Hz, ^3^*J*_HH _= 5.5 Hz, 1H, O_2_C-CH-C*H*(α)), 2.83 (dd, ^2^*J*_HαHβ_ = 12.5 Hz, ^3^*J*_HH _= 7.0 Hz, 1H, O_2_C-CH-C*H*(β)), 1.22 (t, ^3^*J*_HH_ = 7.3 Hz, 12H, N-CH_2_-C*H_3_*); ^13^C-NMR δ 6.64, 31.97, 51.94, 56.16, 118.08, 133.45, 135.84, 181.74; Anal. calcd. for C_14_H_28_N_4_O_2_: C 59.12, H 9.92, N 19.70; found: C 58.92, H 9.73, N 19.45. 

## 4. Conclusions

Nine new tetraethylammonium-based amino acid chiral ionic liquids (CILs) have been synthesized and characterized. All of them showed lower decomposition temperatures but were stable up to 210 °C. By changing the anions, the thermal stability of the CILs is slightly affected due to the configurational and functional groups present in the structure. Although these salts were thermally less stable, they showed much improved viscosity and ionic conductivity compared to similar AAILs. From the trend found in this study, cation structure, ion mobility, *van der Waals* and H–bonding interaction influenced the viscosity and ionic conductivity. Some of these new CILs showed higher ionic conductivity than similar AAILs reported until now. To our knowledge, this paper is the first to report the existence of tetraethylammonium-based amino acids CILs having a high ionic conductivity. These finding should be useful for designing suitable CILs for specific application such as in chiral discrimination, biocatalysis and electrochemical field as electrolyte.
